# mHealth Apps for Dementia, Alzheimer Disease, and Other Neurocognitive Disorders: Systematic Search and Environmental Scan

**DOI:** 10.2196/50186

**Published:** 2024-07-03

**Authors:** Suad Ali, Hira Alizai, Delal Jemal Hagos, Sindy Ramos Rubio, Dale Calabia, Penelope Serrano Jimenez, Vinuu Aarif Senthil, Lora Appel

**Affiliations:** 1 Faculty of Health York University Toronto, ON Canada; 2 Knowledge, Innovation, Talent, Everywhere OpenLab University Health Network Toronto, ON Canada; 3 Women's Brain Health Initiative Toronto, ON Canada; 4 Michael Garron Hospital Toronto, ON Canada

**Keywords:** dementia, Alzheimer disease, mHealth, mobile health, apps, lifestyle behaviors, mobile phone

## Abstract

**Background:**

Lifestyle behaviors including exercise, sleep, diet, stress, mental stimulation, and social interaction significantly impact the likelihood of developing dementia. Mobile health (mHealth) apps have been valuable tools in addressing these lifestyle behaviors for general health and well-being, and there is growing recognition of their potential use for brain health and dementia prevention. Effective apps must be evidence-based and safeguard user data, addressing gaps in the current state of dementia-related mHealth apps.

**Objective:**

This study aims to describe the scope of available apps for dementia prevention and risk factors, highlighting gaps and suggesting a path forward for future development.

**Methods:**

A systematic search of mobile app stores, peer-reviewed literature, dementia and Alzheimer association websites, and browser searches was conducted from October 19, 2022, to November 2, 2022. A total of 1044 mHealth apps were retrieved. After screening, 152 apps met the inclusion criteria and were coded by paired, independent reviewers using an extraction framework. The framework was adapted from the Silberg scale, other scoping reviews of mHealth apps for similar populations, and background research on modifiable dementia risk factors. Coded elements included evidence-based and expert credibility, app features, lifestyle elements of focus, and privacy and security.

**Results:**

Of the 152 apps that met the final selection criteria, 88 (57.9%) addressed modifiable lifestyle behaviors associated with reducing dementia risk. However, many of these apps (59/152, 38.8%) only addressed one lifestyle behavior, with mental stimulation being the most frequently addressed. More than half (84/152, 55.2%) scored 2 points out of 9 on the Silberg scale, with a mean score of 2.4 (SD 1.0) points. Most of the 152 apps did not disclose essential information: 120 (78.9%) did not disclose expert consultation, 125 (82.2%) did not disclose evidence-based information, 146 (96.1%) did not disclose author credentials, and 134 (88.2%) did not disclose their information sources. In addition, 105 (69.2%) apps did not disclose adherence to data privacy and security practices.

**Conclusions:**

There is an opportunity for mHealth apps to support individuals in engaging in behaviors linked to reducing dementia risk. While there is a market for these products, there is a lack of dementia-related apps focused on multiple lifestyle behaviors. Gaps in the rigor of app development regarding evidence base, credibility, and adherence to data privacy and security standards must be addressed. Following established and validated guidelines will be necessary for dementia-related apps to be effective and advance successfully.

## Introduction

### Background

Dementia, a condition with global impact and no current cure, has led to a rise in mobile health (mHealth) apps catering to individuals and caregivers [1-3]. These apps offer features such as location tracking [4], medication reminders [4], education [3], support for caregivers [3], and planning and information sharing across one’s circle of care including with health care providers [5]. Some also focus on screening and diagnostic capabilities, with the ability to analyze large amounts of personal data (eg, changes in voice) that would not have been possible to collect previously [6].

At the same time, research suggests that lifestyle behaviors such as physical activity, sleep, stress, mental stimulation, diet, and social interaction play a crucial role in dementia prevention [7]. Research shows that engaging in these behaviors can significantly decrease the risk of dementia. A recent meta-analysis showed that adults in their 40s who participated in physical activities had a considerably decreased risk of dementia years later [8]. One study found that increased levels of stress and exposure to >2 stressful life events significantly increased the risk of all-cause dementia, while levels of neuroticism caused a higher risk of dementia and Alzheimer disease [9]. Another study found that adults participating in mentally stimulating leisure activities had a significantly decreased risk of developing dementia and other cognitive impairments later in life [10]. According to a random-effect model, individuals with sleep disorders had a higher likelihood of having dementia than those with regular sleep participants [11]. Diet has also been found to be a lifestyle behavior of significance, with one study finding that higher adherence to a Mediterranean diet is adversely related to cognitive decline, dementia, or Alzheimer disease [12]. Similarly, a meta-analysis evaluating social relationships and their impact on the risk of dementia found that social engagement helps prevent dementia. These relationships act as a stress buffer and source of information that help develop positive health behaviors and optimal use of health services [13].

In addition, there is a mounting body of research associating sensory loss and dementia, recognizing a connection between an individual’s vision, hearing, dual sensory (vision and hearing) impairment, smell, and touch with their cognitive health [14,15]. Fischer et al [16] have observed an association between the impairment of the auditory, visual, and olfactory faculties and cognitive decline in older adults. This study suggests that hearing loss and cognitive decline may be magnified by several probable mechanisms including social isolation, a lifestyle behavior associated with dementia risk [16,17]. mHealth apps show potential in supporting the maintenance of users’ sensory health, as observed in a successful pilot that used a mobile app to support electronic devices in the implementation of a home-based cognitive-multisensory-physical exercise program for participants with dementia [18]. These findings demonstrate the potential of exploring if and how dementia-related apps address sensory decline to maintain users’ brain health and prevent cognitive decline.

Currently, there are many popular apps that focus on these lifestyle behaviors and have gained tremendous popularity including MapMyRun that focuses on tracking physical activity and MyFitnessPal, another fitness app focused on tracking diet and calorie intake [19,20]. Although these apps address a number of the lifestyle behaviors (mentioned in the previous paragraphs) that have been linked to the likelihood of developing dementia, few apps have been designed to target these functions specifically for persons with or at risk of developing dementia. Given the movement toward designing mHealth apps to promote healthy lifestyle behaviors (eg, improved diet, exercise habits, and memory-training games), these apps could be leveraged to promote such behaviors earlier in life, with the hopes that this can prevent or delay the onset of dementia or manage symptoms once they present [21,22]. Understanding the breadth of what is available in terms of which lifestyle behavior or combination of behaviors that currently available mHealth apps leverage presents an opportunity to create mHealth apps to address existing gaps and needs for targeted populations.

However, as commercially available smartphone apps can be developed and published by anyone, it is essential to understand which of these are evidence-based so that the public may safely use these apps as a tool for dementia education, prevention, or overall well-being promotion [23]. Since these products may also be eligible for medical device accreditation, or at the very least are considered mHealth or wellness products, we need to understand how they are being marketed, what security and privacy measures are in place to protect their users, and whether the information or tools provided are evidence-based [23]. In particular, these privacy and transparency factors must be strongly considered when leveraging existing or new health apps for persons with dementia or when marketing a certain app as a tool for the prevention of dementia or promotion of well-being. Older adults were found to express concern for their personal information getting hacked while using technology, leading to a hesitancy toward its use [24]. In the case of people with dementia, their personal health information gathered by mHealth apps is at greater risk of being breached [25]. Specifically, many app users with dementia live with impaired cognitive capacity that may interfere with their ability to understand the details of an app’s privacy policies, which are usually presented in confusing or complex language [25].

### Objective

Our objective with this systematic search and environmental scan was to map out the landscape of smartphone apps intended for use by the general public relating to dementia prevention and its risk factors. We have reported on the breadth and depth of available dementia-related mHealth apps and importantly identified gaps that could be addressed by future apps.

## Methods

### Systematic Search and Environmental Scan

This study was conducted according to PRISMA (Preferred Reporting Items for Systematic Reviews and Meta-Analyses) guidelines [26]. Using selected search terms, available mHealth apps directed at dementia and Alzheimer disease and other neurocognitive symptoms were searched for through a systematic search of mobile app stores and supplemented by an environmental scan of peer-reviewed and gray literature. The environmental scan allowed for the identification of a larger subset of relevant apps from external resources to examine the current state of existing mHealth apps directed at dementia and Alzheimer disease and other neurocognitive symptoms [27]. Apps were then screened for eligibility using inclusion and exclusion criteria. Those apps that met the inclusion criteria were then systematically evaluated using an extraction framework developed for this study, available in Multimedia Appendix 1.

### Search Strategies

A systematic search of mHealth apps was conducted by the research team from October 19, 2022, to November 2, 2022. The search terms *Dementia*, *Alzheimer’s*, and *Mild Cognitive Impairment* OR *Cognitive Decline* were entered into the 4 most popular mobile app stores in North America: App Store (Apple), Google Play store, Samsung store, and Microsoft store. These terms were used to identify apps available in these stores that were directed at dementia and Alzheimer disease and other neurocognitive symptoms, with the research team recording up to the first 100 app results as they were generated.

In addition to app stores, the following 3 data sources were searched:

1. The websites of recognized dementia and Alzheimer associations and advocacy groups across North America that listed any recommended or advertised mobile apps: Alzheimer’s Association, Alzheimer’s Family Association, Alzheimer’s Disease International, Alzheimer Society of Canada, Alzheimer Society of Simcoe County, Dementia Society of Canada, National Aphasia Association, and The Ontario Caregiver Association
2. The top 100 article results and listed relevant mobile apps for the search query "Top <dementia/ Alzheimers/ MCI/MCD> apps" on the Google search engine
3. Peer-reviewed meta-analyses, systematic reviews, and scoping reviews on the topic of "Dementia OR Alzheimers OR MCI/MCD and mobile app"

Once duplicates were removed, HA, DJH, SRR, DC, PSJ, and VAS independently screened the apps in pairs based on our inclusion and exclusion criteria, and conflicts were resolved by a third reviewer among them.

### Eligibility Criteria

Apps that indicated any relation to dementia and Alzheimer disease or other neurocognitive symptoms in either title or description were included (eg, dementia-related news, tips for caregivers, and reminder systems). Furthermore, apps available on either the App Store, Google Play, Samsung Galaxy, or Microsoft app stores or a combination of any of the above were included. Textbox 1 defines the inclusion and exclusion criteria for this study in detail.

**App eligibility criteria.**

**Inclusion criteria**

- Available in English
- Title or description of app indicated the purpose or audience related to dementia and Alzheimer disease or other neurocognitive symptoms, using information available on app stores, developer websites, or other web sources

**Exclusion criteria**

- Not available in English
- Title, app store description, or other information available on other app stores, developer website, or other web sources did not indicate that app purpose or audience is related to dementia and Alzheimer disease or other neurocognitive symptoms
- Unavailable on an app store during screening phase

### Data Extraction and Charting Framework

A data extraction framework was developed, partially drawing from the study by Giunti et al [28], where the authors conducted a systematic search of app stores for mHealth apps focused on multiple sclerosis, another neurodegenerative condition. Several elements were modified to fit the context of the nature of dementia risk and lived experience with the condition, which both cope with declining cognitive function. This included the addition of lifestyle behaviors associated with dementia risk as a major coding theme, following background research into the area. The data extraction framework was also designed to set a focus on the credibility of reviewed apps, incorporating coding themes to assess sources consulted, currency, and transparency.

Included apps were independently coded by SA, HA, DJH, SRR, DC, PSJ, and VAS in pairs using the extraction framework described in Multimedia Appendix 1, which included elements such as evidence-based and expert credibility, app features, purpose, lifestyle elements of focus, and privacy and security. In addition, the Silberg scale was incorporated into the data extraction framework, serving as an established evaluation scale designed to assess the accountability of web-based health information, which in this study applies to mHealth apps [29,30]. The Silberg scale was also chosen as it allowed for screening mHealth apps without downloading them and instead used information available through app store descriptions, which included intended use and audiences, available features, and privacy practices, along with information from developer websites when available. While all 9 of the Silberg scale’s evaluation points were adapted from the framework by Jeon et al [29], they were separated from their originally assigned categories and reassigned to an appropriate theme under the new framework.

### Data Analysis

The coding process was performed independently by SA, HA, DJH, SRR, DC, PSJ, and VAS in pairs over multiple iterations, with the researchers meeting to discuss connections between the codes and agree on emerging themes at each stage. The final data set was analyzed by examining the responses of the paired coders or, in some instances, the response of a third coder when present to resolve any conflicts. The consolidated extracted data are available in Multimedia Appendix 2. Descriptive statistics were calculated for numeric responses, which included number of downloads and app store rating. Qualitative data were analyzed using thematic analysis.

## Results

### Overview

A total of 1044 apps were found after the initial search for mHealth apps in 4 popular mobile app stores (n=859, 82.27%) and in peer-reviewed literature (n=78, 7.47%). Apps were also found from nonscholarly sources (dementia and Alzheimer association and advocacy websites: n=62, 5.9%; websites accessed through a browser search: n=45, 4.3%). Following this, 402 (38.5%) duplicates were removed, and 642 (61.5%) apps were screened by HA, DJH, SRR, DC, PSJ, and VAS based on our inclusion and exclusion criteria.

**Figure 1:**
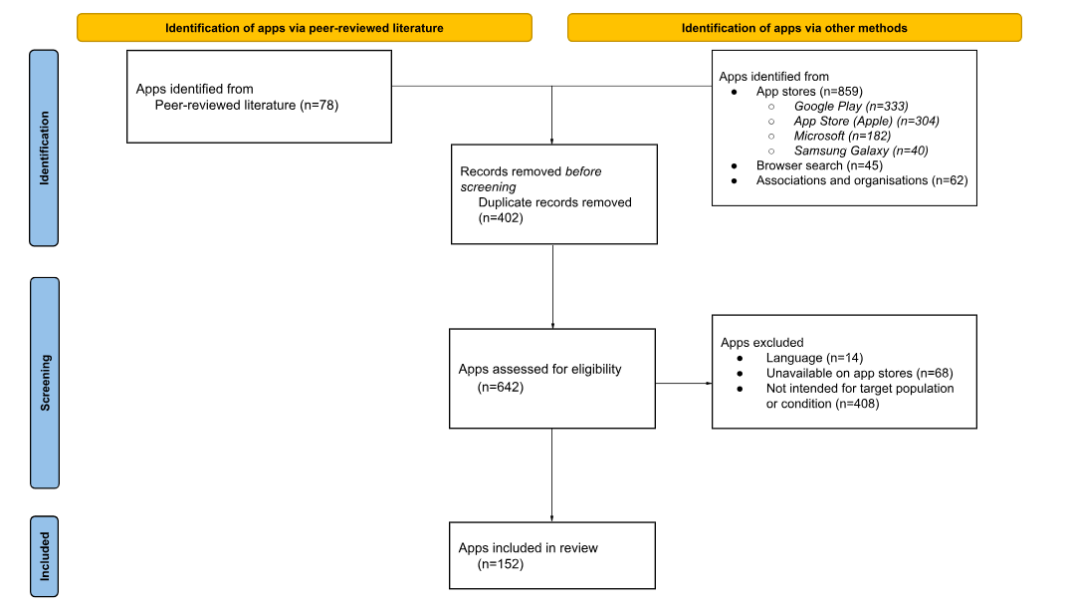
PRISMA (Preferred Reporting Items for Systematic Reviews and Meta-Analyses) flow diagram of study process.

Of these 642 screened apps, 14 (2.2%) were not offered in English, 408 (63.6%) of the apps were determined to not be intended for the target populations or condition, and 68 (10.6%) were unavailable on any of the 4 app stores. With the application of these exclusion criteria, 152 (23.7%) apps remained for full data extraction and charting. The PRISMA flow diagram represented in Figure 1 shows this process at each stage.

### Themes

The following results are presented thematically using the extraction framework details provided in Multimedia Appendix 1, which includes a breakdown of each theme and its objective, along with the operational definition of each variable used within each theme.

#### Evidence-Based and Expert Credibility

At minimum, 120 (78.9%) of the 152 apps assessed did not disclose any expert credibility or use of evidence, such as providing references, citing information sources used, or disclosing that in the process of the app’s development, there was consultation with at least 1 expert, which includes people with lived experience and caregivers. Table 1 illustrates the number of apps that have disclosed these elements.

**Table 1:** Number of apps that satisfy app elements (n=152).

App element		Apps, n (%)
**Variables related to evidence-based and expert credibility disclosed**		
	Consulted with at least one expert	32 (21.1)
	Evidence-based information	27 (17.8)
	Information sources	18 (11.8)
	Funding from a medical or health organization	16 (10.5)
	References	8 (5.3)
	Author credentials	6 (3.9)
**Common mHealth app purposes supported**		
	Education	53 (34.9)
	Reminder features	28 (18.4)
	Diagnostic tools	25 (16.4)
	Habit tracking	13 (8.6)
	Progress tracking	7 (4.6)
	Goal setting	6 (3.9)
	Professional supervision	4 (2.6)
**Lifestyle elements associated with reducing dementia risk**		
	Mental stimulation	59 (38.8)
	Social stimulation	26 (17.1)
	Exercise	10 (6.6)
	Stress management	10 (6.6)
	Eating and nutrition	7 (4.6)
	Sleep	3 (2.0)
**Various elements of transparency disclosed**		
	Application ownership	143 (94.1)
	Author affiliation	20 (13.2)
	Authors credited	11 (7.2)
	Sponsorship	8 (5.3)

Out of the 152 apps, most apps (n=114, 75%) were created by miscellaneous app developers, in other words, developers who were not stated to be associated with any organization or institution. Researchers and health care organizations made up the next 2 largest categories of app creators, observed in 12 (7.9%) and 11 (7.2%) apps, respectively. Members of the public created 8 (5.3%) apps, while medical app developers created 5 (3.3%) apps. Nonprofit non-health care organizations created 2 (1.3%) apps.

#### Purpose

Of the 152 apps, approximately one-third (n=53, 34.9%) were categorized under “Health & Fitness” by app stores. Following this, “Medical” and “Educational” comprised the next 2 largest categories at 40 (26.3%) and 18 (11.8%) apps, respectively. Multimedia Appendix 3 presents the wide range of categories assigned by 4 app stores and the number of apps tagged under each category.

Table 2 illustrates the wide range of intended users of the assessed apps. Note that some apps were observed to have targeted >1 group of users. Of the 152 apps, 105 (69%) apps were intended for people with dementia, making them the most frequently targeted audience. Informal caregivers at 79 (52%) apps and the general public (ie, people without neurocognitive symptoms) at 65 (42.8%) apps comprised the next 2 largest groups.

**Table 2:** Intended app users, up to 3 groups per app (n=152).

Intended user group	Apps, n (%)
People with dementia	105 (69.1)
Informal caregivers	79 (52)
General public	65 (42.8)
Friends or family (noncaregivers)	38 (25)
Clinicians or other providers	27 (17.8)
Dementia Advocates	3 (1.9)
Not stated	2 (1.3)

Of the common mHealth app purposes, the most common was providing education on the health topic (ie, dementia), applicable among 53 (34.9%) out of the total 152 apps assessed. As illustrated in Table 1, the least common was providing professional supervision to users, applicable among 4 (2.6%) apps.

#### Lifestyle Elements of Focus

Of the lifestyle elements important for the prevention of dementia, the most common category was mental stimulation, addressed by 59 (38.8%) out of the total 152 apps assessed. The least common element was sleep, addressed by 3 (2%) apps. Table 1 illustrates these findings. Meanwhile, 65 (42.8%) apps did not focus on any of the lifestyle elements. In addition, only 6 (3.9%) apps addressed sensory health. Table 3 also presents the number of modifiable lifestyle behaviors addressed by the included mHealth apps.

**Table 3:** Number of lifestyle behaviors of focus per app (n=152).

How many lifestyle behaviors does an app focus on?	Apps, n (%)
0	65 (42.8)
1	67 (44)
2	12 (7.9)
3	4 (2.6)
4	2 (1.3)
5	1 (0.6)
6	1 (0.6)
7	0 (0)

#### App Features

Observed in 57 (37.5%) out of the 152 assessed apps, providing communication avenues with various parties was the most frequently observed app feature, while 45 (29.6%) apps incorporated game-based elements. The least embraced feature, found in only 2 (1.3%) apps, was the ability to link to social media.

In addition, there was a lack of mention of features designed for people with dementia such as visuals supplementing texts, voice recognition, and customized accessibility features in the app descriptions.

#### Currency of Apps (as of April 2023)

Of the 152 apps, 136 (89.5%) apps specified their creation date or year and the date of their last update. At the time of analysis in April 2023, only 14 (9.2%) were modified within the previous month.

#### Transparency

Most apps (143/152, 94.1%) had disclosed app ownership, as seen in Table 1, and most apps (132/152, 86.8%) did not disclose any author affiliations or sponsorships or credited any authors or lack thereof.

#### Privacy and Security

**Figure 2:**
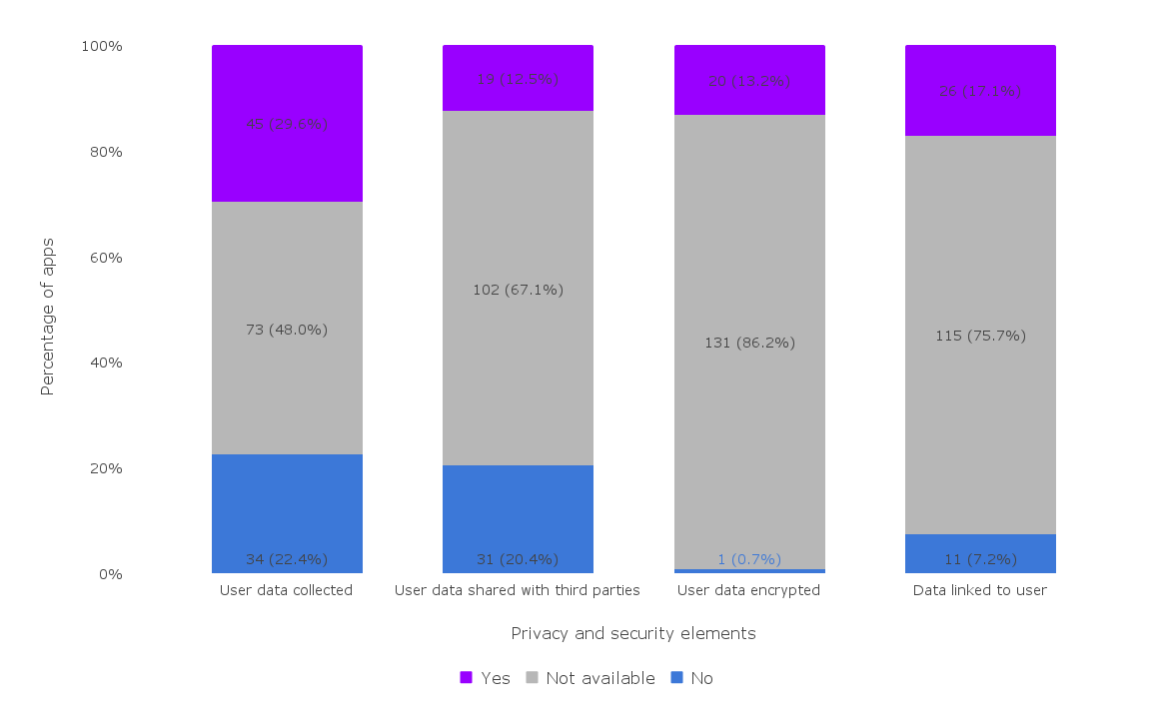
Privacy and security of the assessed apps (n=152).

The most disclosed facet of user privacy and security was whether user data were being collected. This was disclosed by 79 (52%) out of 152 apps, of which 45 (57%) apps collected user data. The vast majority of apps (131/152, 86.2%), however, did not disclose if any user data were encrypted at all, with an average median of 105 (69.2%) apps that did not disclose the privacy and security of their apps. Figure 2 visualizes these observations.

#### App Availability

Of the 152 apps, most apps (n=111, 73%) were free. Free with in-app purchases was the second largest category at 25 (16.4%) apps, followed by 13 (8.6%) premium or paid apps. The smallest category stood with 3 (2%) “freemium” apps, referring to those with basic or limited features available for free download but offering a paid subscription for premium features once installed.

**Figure 3:**
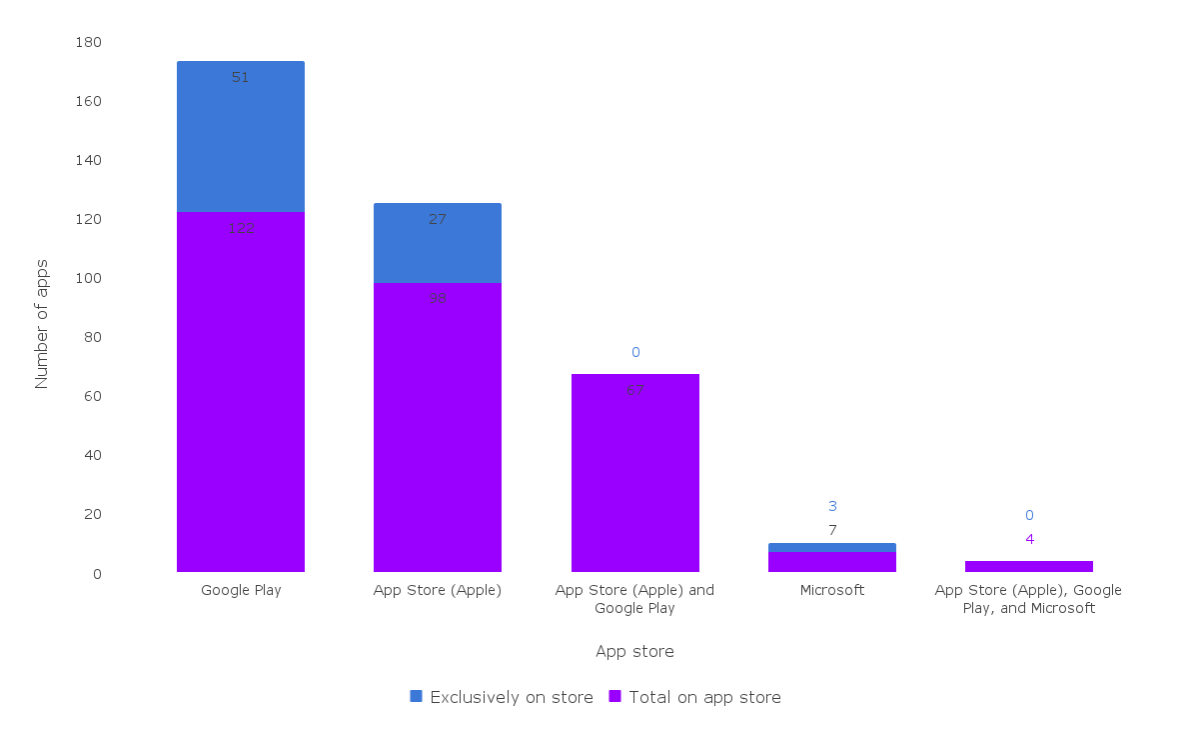
App availability by app store (n=152).

Many apps were available on >1 app store. Figure 3 presents the number of apps available by store, which include the Google Play store, App Store, Microsoft app store, Samsung Galaxy store, and combinations of these.

#### User Reception

Due to the structure of some app stores (ie, certain elements of app information are or are not made available and provided by app stores), 120 (78.9%) out of the 152 assessed apps had no ratings available. The next highest group of app ratings was 4 to 4.9 stars, given to 14 (9.2%) apps. Only 7 (4.6%) assessed apps received a rating of 5 stars.

A total of 77 (50.7%) apps had no download information available due to the structure of some app stores (eg, the App Store did not present this element of app information at the time of charting), while 20 (13.2%) had between 100 and 999 downloads. The next highest number of downloads were those apps with <100 downloads as observed in 19 (12.5%) apps, then between 1000 and 4999 downloads observed in 18 (11.8%) apps. Only 4 (2.6%) apps had between 5000 and 9999 downloads, while 10 (6.6%) apps had between 10,000 and 49,999 downloads. Another 4 (2.6%) apps had between 50,000 and 999,999 downloads.

#### Silberg Scale

As the Silberg scale aims to assess the accountability of web-based health information, with each point representing 1 of 9 elements of accountability disclosed [29,30], a low score suggests that these apps possess gaps in accountability to users. More than half of the apps (84/152, 55.3%) evaluated scored 2 out of 9 Silberg scale points (mean score 2.4, SD 1.0 points), as visualized in Table 4.

**Table 4:** Silberg scale points accrued by apps (n=152).

Silberg scale points	Apps, n (%)
0	4 (2.6)
1	12 (7.9)
2	84 (55.2)
3	33 (21.7)
4	13 (8.6)
5	4 (2.6)
6	1 (0.7)
7	1 (0.7)
8	0 (0)
9	0 (0)

Of the 152 apps, 4 (2.6%) apps did not score any Silberg scale points (ie, received total scores of 0), and none of the included apps achieved a perfect score. The Care4Dementia app totaled to 7 points, forgoing 2 elements of app accountability: crediting the app’s authors and indicating whether an update had been made within the last month (as of the screening and review stages). Meanwhile, the Memory Lane Games app received a score of 6, forgoing the same elements as the Care4Dementia app along with failing to disclose author credentials.

## Discussion

### Principal Findings

This study aimed to assess the scope of mHealth apps intended for use by the general public relating to dementia prevention and its risk factors and to determine any gaps pertaining to factors including accountability of information, addressing modifiable lifestyle behaviors, and privacy and security. The findings of this systematic search and environmental scan of mHealth apps have found that most lack sufficient accountability of information, particularly for products that target health and can store sensitive user information. Most apps assessed did not satisfactorily disclose their evidence base, lacked comprehensive focus on >1 modifiable lifestyle behavior related to reducing dementia risk, and did not disclose adherence to privacy and security measures or established privacy guidelines. However, apps did show promising diversity in its target audiences, as a number of apps were designed for caregivers of people with dementia (and more broadly, people with neurocognitive symptoms); friends and family; clinicians; or the general public (ie, people without neurocognitive symptoms). These gaps highlight opportunities for researchers and clinicians to establish participatory design frameworks for developing rigorous mHealth apps, as the interest in dementia prevention grows and is projected to continue to grow.

In this study, our goal was to describe the breadth and depth of dementia-related mHealth apps to understand whether these apps demonstrate factors such as credibility, privacy, and transparency in order to be leveraged as a tool for the prevention of dementia. In addition, this study sought to explore whether dementia-related apps specifically addressed any of the above-discussed lifestyle behaviors attributed to maintaining brain health. In the following sections, we will report on the state of apps intended for the public relating to the prevention of dementia by the themes identified above.

### Themes

#### Evidence-Based and Expert Credibility

Most apps did not report author credentials, give information on whether peer-reviewed papers or other sources were used, or provide references. When creating apps that address people with dementia, there should be clear guidelines for developers to follow to ensure that a minimum standard is met in terms of how credible the information in the app is, such as including the source of evidence-based information presented [31]. The absence of these factors could impact the overall effectiveness of the app and its uptake by patients or could mislead and misinform individuals looking to use apps to improve their brain health.

Similarly, most assessed apps (114/152, 75%) did not specify the app developer association, meaning their affiliations were not clearly defined. Meanwhile, only a small subset of assessed apps (23/152, 15.1%) had been created by developers categorized as researchers or health care organizations. This reinforces the need for more credibility and reporting of evidence-based information that could benefit users. Furthermore, only a few indicated having consultations with an expert, which included people with dementia or caretakers of people with dementia, whose lived experiences could be strongly leveraged to take input from, as apps are developed to influence behaviors and habits [32]. The lack of consultation with experts [33] or people with lived experience could impact the quality of the app and the information delivered, making these apps less relevant to those who could best benefit from it. These gaps may mislead consumers in the target populations or provide them with misinformation that could further affect their condition onset or prognosis.

#### Purpose

Of the 152 apps assessed, 96 (62.5%) were categorized under “Health & Fitness,” “Medical,” and “Educational,” with most apps intended for persons with neurocognitive symptoms, informal caregivers, and the general public (ie, people without neurocognitive symptoms). Meanwhile, only a small subset of these apps actually provides information pertaining to dementia; offers diagnostics tools; and provides reminder, goal-setting, progress-tracking, or habit-tracking features. The lack of diagnostic tools and ability for professionals to remotely monitor users align with the minimal evidence-based information or clinical credibility available in these apps. There appears to be a gap in what these apps that claim to target persons with dementia (and more broadly, people with neurocognitive symptoms) realistically provide to benefit this population. Education, reminder features, goal setting, and tracking would be beneficial tools to provide through apps not only for the target population but also for informal and formal caregivers as well (ie, apps could help set reminders for medications while providing education on certain habits that individuals can commit to improving their condition) [34].

#### Lifestyle Elements of Focus

Apps were screened for whether they focused on one of the modifiable lifestyle behaviors associated with reducing the risk of developing dementia, which include exercise, sleep, stress management, mental stimulation, eating and nutrition, and social stimulation. More than 2 in 5 apps did not address any lifestyle behavior, while 57.2% (87/152) addressed at least 1. When apps did address modifiable lifestyle behaviors, it was most common to focus on 1 behavior at a time while few addressed a combination of 2 behaviors per app, as demonstrated in Table 1. The lifestyle behavior of focus was mental stimulation; a greater focus on mental stimulation in these apps is in line with the number of apps that had contained game-based elements. This complements the observations of many studies that dementia prevention mHealth apps focused on mental stimulation in maintaining cognitive vitality in healthy individuals and those with mildly impaired cognition, including improved memory and enhanced quality of life [35].

Research suggests that activities addressing these lifestyle behaviors could in fact improve brain health and lower the chances of developing dementia. In a study exploring physical exercise, diet, smoking, alcohol consumption, cognitive stimulation, and social stimulation, Jia et al [36] observed that positively maintaining any one of these lifestyle behaviors contributes to slowing memory decline. Mamalaki et al [37] also observed the individual impacts of diet, physical activity, sleep, and engagement in activities of daily living (including social stimulation) in maintaining cognitive vitality and reducing the risk of developing dementia. Both studies highlighted the combined effects of addressing multiple lifestyle behaviors to further slow cognitive decline, with the upkeep of more behaviors resulting in stronger prevention [36,37]. It is noteworthy to mention that both studies found diet as the modifiable lifestyle behavior with the largest associated effect on maintaining cognitive vitality and reducing the risk of decline. Thus, if apps are to target patients and their caregivers, it would be most useful to use this evidence-based information to allow for habit development and tracking in activities related to exercise, reminders to maintain sensory well-being, or prompts that could structure stress management. In particular, delivering interventions related to the prevention of dementia using the mHealth app format can expand the availability of these products to hard-to-reach populations [38].

#### App Features

Apps were also categorized based on whether they offered game-based elements, communication avenues, and links to social media. These features are attractive and can address feelings of loneliness for patients. While game-based apps can be a therapeutic experience, this population may have difficulty in carrying out the tasks or activities required by some of these game apps. Apps may also be a promising way to address social isolation and loneliness [39], providing a channel for in-app users to connect among themselves or presenting features to link with family, friends [40], or the general public for connection or support.

One subset of the intended users of these apps, that is, persons with dementia, requires specific design features for these apps to be beneficial and accessible. Such features include prompts (eg, redirection prompts while completing an activity), visuals supplementing the text, reminder systems, and voice recognition, along with the option to customize accessibility features because dementia affects each of those with the condition differently [41,42]. If these game-based apps are targeted at patients, they may be inaccessible for them due to impaired cognitive abilities where they cannot complete multistep cognitive tasks, remember information, or answer questions in a short time span as they may be cognitively demanding. These features are clearly lacking, making them less accessible to the target users. Thus, the gap remains that consulting the target populations during the development of these apps to identify useful elements would likely serve as the best use of time and resources to yield a positive impact on patients.

#### Currency of Apps (as of April 2023)

Information on currency, year of creation, and last update date were available for most of the apps. This practice of transparency around app currency is important as new research is released regularly and thus would be important for the consumer to understand when the app itself was created along with the date of the information that the app is based on.

#### Transparency

A minority of apps credited authors to acknowledge their work, reported author affiliation to demonstrate connection to an organization or academic center, or disclosed sponsorship on whether a specific organization provided funding for the development of the app. This results in poor transparency, as it does not provide consumers with the necessary information to identify the basis of the app or disclose whether there is a possible conflict of interest because of a sponsorship, author, or organization affiliations. Moreover, it is crucial to disclose all these pieces of information, as simply reporting sources or authors while failing to report sponsoring organizations obscures acknowledgment of possible bias embedded in the information presented and the intention behind mHealth apps [43,44]. Providing this information to users could add credibility to the app, which could help an individual decide whether or not such an app will serve their needs and whether it is trustworthy and safe to use [23,45]. Furthermore, the availability of this information is crucial to ensure patient safety and suitability on part of professionals who may recommend such mHealth apps for clinical use [23,45]. By contrast, users are less aware of and less preoccupied with considering an app’s evidence base and instead emphasize the quality of its consumer-oriented, practical use such as data use, battery drainage, loading speed, and presence of advertisements when deciding whether to continue its use [43].

#### Privacy and Security

In regard to privacy and security of these apps, most apps did not provide information regarding the collection of user data, data sharing with third parties, user data encryption, or data linking to users. This demonstrates a gap in the security of app-user data, which may be at risk of being compromised due to weak data governance guidelines that are not disclosed clearly or at all. Close attention should be paid to ensuring the privacy and security of mHealth apps, especially when dealing with susceptible target groups such as people living with mild cognitive impairment [46]. One relevant guideline that can be followed is the *mHealth Data Security, Privacy, and Confidentiality: Guidelines for Program Implementers and Policy Makers*, which details best practices and considerations for mHealth app development. These best practices cover the data life cycle stages of data capture and storage, access, transfer, and disposal, as well as address risks associated with operating systems, mobile devices, networks, and mHealth data storage [47]. The *Privacy and Code of Conduct on Mobile Health Apps* developed by the European Commission [48] lays out principles for mHealth app development and function, including considerations for user consent, app security measures, and use of user data for secondary purposes such as research and third-party sharing among others. While these guidelines are not official law or policy, they provide detailed considerations that will assist mHealth app developers in creating and sustaining responsible data governance.

#### App Availability

For apps to be useful, they should be accessible to the targeted population. Commercialization of the apps significantly impacts this, as almost three-quarters of assessed apps were offered for free, while about one-tenth involved a purchase for the basic or the full version of the app. Furthermore, 44.1% (67/152) of the apps were offered both on the App Store and Google Play app store, whereas an exclusive 33.5% (51/152) were offered only on Google Play as opposed to 17.8% (27/152) of apps that were exclusively available on the App Store. Interestingly, only a small number of the apps (7/152, 4.6%) were available on the Microsoft app store, and none were offered on the Samsung Galaxy app store. A needs-based analysis to identify which app features are most useful for patients and caregivers would be appropriately supplemented by identifying the type of smartphones that are accessible to this population.

#### User Reception

Measured by the number of downloads, user reception is important when it comes to measuring meaningful use of apps. While not all apps had information pertaining to their exact number of downloads, 39 (25.7%) out of the 152 assessed apps had <999 downloads, of which 19 (49%) had <100 downloads per app. This illustrates a very low use or download rate of apps that are categorized and are targeted for persons with neurocognitive symptoms.

#### Silberg Scale

Apps were also scored using the Silberg scale, which is an established validated measure to capture the extent of accountability of health information presented on the web, including through mHealth apps [29,30]. An app can score up to a total of 9 Silberg scale points, wherein the higher the total Silberg score, the more the accountability of the information presented by or used to create the app is communicated [49,50]. Meanwhile, a low total Silberg score implies the opposite, suggesting that the app in question does not transparently disclose the credibility of the app’s information, which may therefore lack accountability toward users and the public. Within this study, the mean Silberg scale score of 2.4 (SD 1.0) across all reviewed apps points toward a trend skewed toward low accountability of information used and presented by the apps (ie, in communicating the authorship, attribution, disclosure, and currency of their information). The observed skewed trend of apps with low Silberg scale scores raises concern for a lack of accountability these apps take in disclosing the credibility of their foundations, their reliability, usefulness to targeted users, and the efficacy and effectiveness of their results, if any.

While the apps assessed in this study produced low Silberg scale scores, this does not appear to be unique in this regard, as other nondementia mHealth apps have observed similar findings. Several studies assessing mHealth apps using the Silberg scale found that most apps scored <5 points [29,51-53]. In the case of a study assessing the (then) current state of mHealth apps that addressed postnatal depression, the authors observed a mean Silberg score of 3.0 points and suggested the need for the involvement of health care professionals in developing mHealth apps [51]. In another study, an analysis of obesity management mHealth apps observed a mean score of 4.55 points along with a low percentage of apps disclosing information sources and references [29]. The assessed obesity management apps were found to present poor accountability of the information, highlighting the need to improve the current state of mHealth app information quality. The systematic review of mHealth apps by Feldman et al [53] that had addressed peripartum mood disorders reached a similar conclusion, finding <30% of the assessed apps indicated the use of research for app development. Overall, the authors found that most apps reviewed in their study were of poor quality, as indicated by low Silberg scores, and suggested their poor accountability of information as a hindrance to users in making an informed decision about their quality [53]. Overall, there is a general trend of low Silberg scale scores observed across mHealth apps, which is a gap that requires addressing by developers.

The observations noted in this section point toward the need for app developers and app stores to fill in the gaps when presenting descriptions of the products they are offering. The most significant gaps observed in targeted apps included (1) a lack of prevention-oriented apps addressing >1 modifiable lifestyle behavior (diet, sleep, exercise, stress management, mental stimulation, social stimulation, and sensory health) associated with dementia risk; (2) an absence of apps created using evidence-based information and credible authors and an absence of transparent disclosure of these sources; (3) inadequate rigor of general mHealth data privacy and security procedures and a complete lack of disclosed specific guidelines created for people with cognitive impairment; and (4) a lack of evidence-based, usable app features designed for the needs of people with declining or limited cognitive abilities.

### Limitations

While efforts were made to make this study systematic and comprehensive, several limitations should be acknowledged. This study used PRISMA with the goal of adhering to rigorous standards and to thoroughly structure its data search, screening, and extraction. However, PRISMA guidelines are intended for systematic reviews and meta-analyses evaluating peer-reviewed literature, contrasting with this study’s environmental scan component along with its evaluation of mobile apps. In addition, a limited selection of app stores were searched from the available pool of North American app stores, and reviewed apps had to be available in English, and thus, this study may not have captured the entirety of dementia-related apps available on the global market. While peer-reviewed journal articles, browser searches, and dementia and Alzheimer association and organization websites were initial sources for identifying available apps, the search using these sources was not exhaustive. There are also additional search terms that could have been used to widen the search results such as "memory," "brain training," and "cognit*." As a result, there is a possibility that some potential apps for identification may have been missed, and we recommend taking this into account for future research.

Due to the dynamic nature of app stores, some apps included in this study’s final review have been pulled out of app stores since the time of analysis. In addition, the structural differences among the 4 app stores resulted in some details being available on 1 or some app stores (ie, number of app downloads, user data collection, sharing of user data with third parties, user data encryption, and linking data to user) and not on other app stores. Because of this, some data points were extracted as “N/A” and do not present a full picture of the variable.

Apps were not downloaded, meaning that features and functionality could not be assessed to the fullest extent. Thus, relying on app store descriptions as the primary source for information, supplemented with other sources available on the web, we could not extract the data regarding the full extent of app features, purposes, audiences, and other elements in the screening and data extraction phases. Furthermore, some descriptions encountered provided only scarce information, impacting the scope of data extraction for individual app screeners at times. However, second- and third-screener corroboration was conducted in response to this and ensured complete data extraction.

Finally, in the process of screening for mHealth apps, we encountered a challenge in accurately distinguishing between those targeted for users with mild cognitive impairment and dementia due to the inherent complexities and overlapping characteristics of these conditions. Recognizing the importance of this distinction in clinical contexts, it is crucial to acknowledge that our screening criteria erred on the side of inclusivity, resulting in a broader scope of apps included in our review. While this may initially seem to widen the spectrum of included apps, it inherently contributes to a more comprehensive and nuanced report of the current landscape of available apps. By encompassing a broader range of users experiencing neurocognitive symptoms, our study provides a holistic view of the mHealth app landscape, offering valuable insights into potential digital interventions for individuals at varying stages of cognitive decline. This approach enhances the practical relevance of our findings and acknowledges the diverse needs of users in the evolving field of digital therapeutics.

### Conclusions

As the prevalence of dementia continues to grow, so does the burden of disease on patients, caregivers, the health system, and the greater community. This could be combated by leveraging mHealth apps to address and mediate the symptoms and effects of these diseases. Specifically, the literature suggests addressing a number of modifiable lifestyle behaviors as an effective way to improve brain health. In order to address these behaviors through mHealth apps, the apps should not only be accessible in terms of availability but also offer features suited for individuals living with declining or impaired cognitive vitality. Furthermore, these apps should be transparent in the foundation of information, its evidence base, and the involvement of professionals or people with lived experience in its development. Thus, providing app designers and developers with a guideline for what is required and necessary to fulfill the needs of patients and caregivers, along with minimal standards for what an app must have to be appropriate for this susceptible population, would be ideal. Such a guideline would be instrumental in leveraging mHealth apps to maintain cognitive vitality, socially connect, and provide therapeutic avenues for persons with dementia (and more broadly, those with neurocognitive symptoms) or their caregivers.

## Appendix

Multimedia Appendix 1 Extraction framework, organized by themes.

Multimedia Appendix 2 Full set of extracted and coded data from included mobile health apps (n=152).

Multimedia Appendix 3 Apps by app store categories (n=152).

## Abbreviations

mHealth: mobile health
PRISMA: Preferred Reporting Items for Systematic Reviews and Meta-Analyses
