# Supplementary material for: mHealth Apps for Dementia, Alzheimer Disease, and Other Neurocognitive Disorders: Systematic Search and Environmental Scan
Source: JMIR Mhealth Uhealth. 2024 Jul 3;12:e50186. doi: 10.2196/50186 (PMC11255539; doi:10.2196/50186)
Supplement: Multimedia Appendix 1 [file mhealth_v12i1e50186_app1.docx]

| Theme | Theme Objective | Variable | Operational Definition |
| --- | --- | --- | --- |
| **Evidence- based and expert credibility** |  |  |  |
|  | Evaluates validity of sources consulted to create app itself | Author credentials | App discloses academic background, work experience, or history of published academic papers of individuals involved in developing content |
|  |  | Information sources given | App discloses use of peer-reviewed papers in academic journals, white papers, websites/webpages, books, blogs, lectures, best practice guidelines, databases, government standards or policies, interviews, or archives used to develop app or support its content or use |
|  |  | References given | App discloses links/hyperlinks or citation reference of information sources used to develop app or support its content or use, if app does indicate use of information source(s) |
|  |  | Disclosure of evidence-based information used | App discloses if sources that have undergone rigorous academic/scientific process were used for its development- specifically peer-reviewed articles published in academic journals, or best practice guidelines sourced from governmental sources or medical/healthcare organizations. These sources exclude all other information sources including those published in blogs, books, magazines, websites/webpages, videos, and others unless otherwise disclosed as peer-reviewed or having undergone academic/scientific process |
|  |  | Indication of expert(s) consultation used | App discloses if medical professionals, allied health professionals, researchers/academics, people with dementia, or caretakers of people with dementia were consulted for development |
|  |  | Funding by a medical/healthcare organization | App discloses if development and/or maintenance was funded by an individual hospital, hospital network, medical clinic, rehabilitation facility, medical laboratory, long-term care home, surgical center, urgent care center, telehealth organization, or other medical/healthcare organization |
|  |  | App creators | App discloses names of the specific individuals involved in its development |
| **Purpose** |  |  |  |
|  | Evaluates intended app use and/or outcomes | App store category/tag | Categorization provided by app store where the app has been sourced from. Categories/tags include Fitness, Medical, Games, and more |
|  |  | Intended users | Audience(s) for whom the app’s authors and/or sponsors developed the app for.  Intended user categories are: People with dementia; Informal caregivers; General public (i.e., people without neurocognitive symptoms); Friends or family who do not act in a caregiving capacity; Clinicians and other care providers; dementia/Alzheimer’s advocates; Policy-makers, or; Not available. Up to three different intended user categories can be extracted |
|  |  | User education | App can be used to support user’s understanding and knowledge of what dementia is, its causes, methods of diagnoses, treatment, current state of research, lived experience with dementia, caretaking for a person with dementia, coping with dementia, and/or methods for prevention. Achieved using text, visuals, auditory-only presentation, audiovisual presentation, or interactive graphics |
|  |  | User diagnosis | App can be used to diagnose possibility of user developing dementia, or diagnose stage of dementia that user has reached through use of tests or other validated assessment tools |
|  |  | User reminder | App can be used to remind user to complete activities to support their well-being including chores to complete and their instructions, completing hygiene, time to sleep, what to eat and timings, medicine to take along with their instructions and timings. Can also be used to assist users with dementia with recognizing themselves or loved ones, and/or recalling memories of the past |
|  |  | User goal-setting | App can be used to set goals to support user’s well-being including chores to complete, sleeping a specified duration of time, going to sleep and/or waking up at a specified specific time, taking a nap or not, meeting nutrition goals or number of meals eaten in a day, taking one’s medicines, completing to-do tasks, or completing recreational activities in a specified period of time. Can also be used to set gaming goals (ex. completing a certain number of levels) for those apps supporting games/game-based elements |
|  |  | User goal progress- tracking | App can be used to keep track of progress of user’s goals, notify whether they have been reached during user-set timeframe, and/or provide user with steps to stay on track of reaching goals |
|  |  | User habit-tracking | App enables users to record and review their behaviors through manual user input, including sleep duration and timings over a period of time, periods where user demonstrated cognitive decline, or periods of app non-use. Can capture this information through in-app prompts. Can allow automatic recording of user behaviors via smartphone sensors, including steps taken, periods of physical inactivity, user heartbeat, and user’s geographic location and time spent in one place. |
|  |  | Professional supervision | App supports remote supervision of user behaviors/activity, responses, or lack thereof by health professionals (ex., physicians, nurses, surgeons, pharmacists, social workers, behavioral therapists, dietitians, and others) which can be set and/or followed-up remotely or during in-person appointments |
| **Lifestyle element(s) of focus** |  |  |  |
|  | Evaluates lifestyle behaviors associated with dementia risk | Exercise | App supports user’s physical activity through user education achieved by presenting written or visual demonstrations of stretches or exercises, activity goal-setting or tracking, and/or activity reminders sent to user via app. Can also educate user on benefits of exercise, and consequences of being sedentary |
|  |  | Sleep | App supports user sleep habits through goal-setting sleep duration or sleep times, reminders, alarms, and/or sleep tracking. Can also educate user on benefits of sleep to them generally and/or in the context of dementia. |
|  |  | Stress management | App supports users in identifying sources of stress and methods, determining and carrying out methods to address and reduce stress in their life, and enabling consistent use of these identified stress-reducting methods (ex. app sending regular check-ins, setting goals, or providing feel-good reminders). Can also educate user on benefits of stress management and consequences of ignoring stress management. |
|  |  | Mental stimulation | App supports user perform in-app activities that challenge user cognitively, such as word-based games, avatars on missions, or object matching. Can also support user in performing activities outside of app environment that require user’s mental engagement beyond daily norm. Can also educate user on benefits of maintaining mental stimulation and consequences of ignoring this behaviour. |
|  |  | Eating and nutrition | App supports user assess fulfilment of nutritional requirements and/or enables meeting these requirements, either by themselves, through professional supervision, caretaker assistance, and/or loved ones’ involvement. Can also assess whether app enables user to track, receive reminders for, and set goals for meal-times. Can also provide eating and nutrition education, including recipes and instructional visuals, explaining impacts of nutrients on body, and/or how eating changes with dementia |
|  |  | Social stimulation | App supports user in socializing with other app-users, accommodates socialization with the greater public, and/or enables in-app connection of users with loved ones for the purpose of recreational communication or revisiting memories. Socialization can be conducted through text-based (ex. chat forums, direct messages, group chats), oral (ex. voice messages, phone calls), auditory (ex. sharing music or sounds), or visual (ex. sending pictures or videos) means for the purpose of pleasure and/or support |
|  |  | Sensory health | App supports maintenance of user’s sense of hearing, taste, sight, touch, or smell. Sensory health can be supported through undertaking habits that maintain sensory health and/or avoiding those that contribute to sensory loss |
| **App features** |  |  |  |
|  | Evaluates app elements that support intended purpose, and shape user experience | Games/game- based elements | App contains features that engage users in playful activities using visuals and/or audio, such as puzzles, matching/pairing/finding objects on screen, word association, and math quizzes, amongst others. These elements implement a points or awards system, user progression to new levels, unlock new activities, provide feedback, and/or indicate the upholding of mental stimulation or social interaction |
|  |  | Communication with professionals | App contains features that enable user-to-caregiver, user-to-clinician, or user-to-counsellor communication within app. This can take place through direct messages, video calls, or leaving comments/notes and replies on in-app charts |
|  |  | Communication with app-users | App contains features that enable in-app user-to-user communication. This can take place through direct messages, chatrooms, posting reviews, video calls, or social media-style forums |
|  |  | Communication with friends and/or family | App contains features that enable user-to-relative, user-to-friend, or user-to-support person communication within app. This can take place through direct messages, video calls, or private group forums customized for app-user’s personal network |
|  |  | Link to social media | App includes elements/settings that allowed for connection of app users’ profiles to established social media platforms in order to communicate with other users or members of the public and/or to broadcast progress |
| **Currency** |  |  |  |
|  | Focuses on how often apps are maintained and updated by authors/developers | Application modified in the previous month | App has undergone an update from developers within past 30 days |
|  |  | Creation/last modification date specified | Month and year (optional: date) when app was first launched/published/offered on app store is disclosed |
|  |  | Year of creation | Year when app was first published/launched/released on app store |
|  |  | Date of latest update | Month and year (optional: date) app was last updated by developers on app store |
| **Transparency** |  |  |  |
|  | Evaluates if and to what extent app author and/or sponsorship details are disclosed | Authors credited | App acknowledges individuals involved in creating app’s content for their work |
|  |  | Author affiliation | App discloses employment and/or academic connections of app authors to organizations, such as universities, research institutions, or corporate research centers |
|  |  | Application ownership disclosed | App developer (either a company or individual) offering the product (i.e., app) on app store is specified |
|  |  | Sponsorship disclosed | Involvement of an organization of any kind (ex. academic, corporate, medical facility) that has provided funding for app’s development |
| **Privacy/ security** |  |  |  |
|  | Evaluates user data privacy and security | User data collected | App store description explicitly indicates, using a clear subheading, that data inputted by user and/or generated through app’s use is or is not collected, or if this information is unavailable |
|  |  | User data shared with third parties | App store description explicitly indicates, using a clear subheading, that user data inputted by user and/or generated through app’s use is or is not distributed to third-party organizations |
|  |  | Encrypted user data | App store description explicitly indicates, using a clear subheading, that user data is or is not encrypted, or if this information is unavailable |
|  |  | Data linked to user | App store description explicitly indicates, using a clear subheading, that data inputted by user and/or generated through app’s use is or is not connected to user, or if this information is unavailable |
| **App availability** |  |  |  |
|  | Evaluates public’s initial ease of access in downloading app | Commercialization | Offering of app through mandatory payment for download, voluntary payments in exchange for enhanced features, or no payments at all for download and use of app. Assessed as app availability for free, premium (i.e., only available for download with payment), freemium (i.e., a free but limited version is available for users with the option of paying for the full, enhanced version), or free with in-app purchase(s) |
|  |  | Apps availability on more than one app store | App is available on more than one app store amongst Google Play store, iOS store, Microsoft store, or Samsung Galaxy store |
| **User reception** |  |  |  |
|  | Evaluates quantitative indicators of the public’s acknowledgement and interest in app | App store rating | Number of stars app has received by users or non-user members of the public, if this metric is available on app store website, developer website, or other Internet source |
|  |  | App store downloads | Number of times app has been downloaded from app store, if this metric is available on app store website, developer website, or other Internet source |
