# Supplementary material for: mHealth Apps for Dementia, Alzheimer Disease, and Other Neurocognitive Disorders: Systematic Search and Environmental Scan
Source: JMIR Mhealth Uhealth. 2024 Jul 3;12:e50186. doi: 10.2196/50186 (PMC11255539; doi:10.2196/50186)
Supplement: Multimedia Appendix 3 [file mhealth_v12i1e50186_app3.docx]

| Category | Number of apps^a^ | Example |
| --- | --- | --- |
| Health & Fitness | 53 | [Dementia Clock](https://play.google.com/store/apps/details?id=com.wearingthegreen.pc.otdementiaclock) |
| Medical | 40 | [Pain Rating Scales](https://play.google.com/store/apps/details?id=com.etz.painassessment) |
| Educational | 18 | [Alzheimer’s School](https://play.google.com/store/apps/details?id=com.kpsoftsolns.alzheimersworld) |
| Lifestyle | 9 | [Alzheimers be my friend](https://play.google.com/store/apps/details?id=com.Alzheimer.dashboard.www.android61a419ddc4c4d) |
| Puzzle | 5 | [OnCortex](https://play.google.com/store/apps/details?id=com.AnzcadeGames.OnCortex) |
| Games | 4 | [Brain training for Dementia](https://play.google.com/store/apps/details?id=mywowkr.TurnCardUp&hl=en_CA&gl=US) |
| Offline | 4 | [Brain training for Dementia](https://play.google.com/store/apps/details?id=mywowkr.TurnCardUp&hl=en_CA&gl=US) |
| Single Player | 4 | [Games for visually impaired](https://play.google.com/store/apps/details?id=com.oxothuk.puzzlefeedblind) |
| Tools | 4 | [Dementia Counter version 3.1](https://play.google.com/store/apps/details?id=appinventor.ai_adams1stephen.Dementia_Counter&hl=en_CA&gl=US) |
| Books & References | 3 | [Iridis](https://play.google.com/store/apps/details?id=com.SpaceAppliedTechnology.Iridis&hl=en_CA&gl=US) |
| Casual | 3 | [Games for Visually Impaired](https://play.google.com/store/apps/details?id=com.oxothuk.puzzlefeedblind) |
| Board | 2 | [Alzheimer help](https://play.google.com/store/apps/details?id=kaipnru.game.ped) |
| Entertainment | 2 | [AmuseIT](https://play.google.com/store/apps/details?id=nz.amuseit.amuseitquiz&hl=en_CA&gl=US) |
| Social Networking | 2 | [MemoryHome](https://play.google.com/store/apps/details?id=com.lannoo.memoryhome&hl=en_CA&gl=US) |
| Abstract | 1 | [KettleMind- Competitive Brain](https://play.google.com/store/apps/details?id=com.happyadda.kettlemind) |
| Brain Teasing | 1 | [KettleMind- Competitive Brain](https://play.google.com/store/apps/details?id=com.happyadda.kettlemind) |
| Brain Training | 1 | [KettleMind- Competitive Brain](https://play.google.com/store/apps/details?id=com.happyadda.kettlemind) |
| Competitive Multi-player | 1 | [Concentration: Match Game](https://play.google.com/store/apps/details?id=com.brandedbrothers.memorymatch&hl=en_CA&gl=US) |
| Memory | 1 | [Concentration: Match Game](https://play.google.com/store/apps/details?id=com.brandedbrothers.memorymatch&hl=en_CA&gl=US) |
| Multi-player | 1 | [Concentration: Match Game](https://play.google.com/store/apps/details?id=com.brandedbrothers.memorymatch&hl=en_CA&gl=US) |
| News & Weather | 1 | [Alzheimer’s News](https://apps.microsoft.com/store/detail/alzheimers-news/9NBLGGH2VC19) |
| Pair Matching | 1 | [Concentration: Match Game](https://play.google.com/store/apps/details?id=com.brandedbrothers.memorymatch&hl=en_CA&gl=US) |
| Personalization | 1 | [AMOM - Elderly Alzheimer Demen](https://play.google.com/store/apps/details?id=com.amom) |
| Realistic | 1 | [Concentration: Match Game](https://play.google.com/store/apps/details?id=com.brandedbrothers.memorymatch&hl=en_CA&gl=US) |
| Reference | 1 | [Alzheimer’s Assoc Meetings](https://apps.apple.com/ca/app/alzheimers-assoc-meetings/id1466083985) |
| Social | 1 | [MemoryHome](https://play.google.com/store/apps/details?id=com.lannoo.memoryhome&hl=en_CA&gl=US) |
| Trivia | 1 | [Alzheimer Training](https://play.google.com/store/apps/details?id=appinventor.ai_bruno_jogos0000.PAP) |
| Word | 1 | [Crossword Game for Seniors](https://play.google.com/store/apps/details?id=wordgame.seniors.alzheimer.dementia.braintraining&hl=en_CA&gl=US) |
| ^a^During data extraction, it was observed that app stores tagged some apps under more than one category. | | |
